# A MT1-MMP/NF-κB signaling axis as a checkpoint controller of COX-2 expression in CD133(+) U87 glioblastoma cells

**DOI:** 10.1186/1742-2094-6-8

**Published:** 2009-03-09

**Authors:** Borhane Annabi, Carl Laflamme, Asmaa Sina, Marie-Paule Lachambre, Richard Béliveau

**Affiliations:** 1 Laboratoire d'Oncologie Moléculaire, Département de Chimie, Centre de Recherche BIOMED, Université du Québec à Montréal, Quebec, Canada; 2 Laboratoire de Médecine Moléculaire, Department of Neurosurgery, CHUM and UQAM, Montreal, Quebec, Canada

## Abstract

**Background:**

The CD133(+) stem cell population in recurrent gliomas is associated with clinical features such as therapy resistance, blood-brain barrier disruption and, hence, tumor infiltration. Screening of a large panel of glioma samples increasing histological grade demonstrated frequencies of CD133(+) cells which correlated with high expression of cyclooxygenase (COX)-2 and of membrane type-1 matrix metalloproteinase (MT1-MMP).

**Methods:**

We used qRT-PCR and immunoblotting to examine the molecular interplay between MT1-MMP and COX-2 gene and protein expression in parental, CD133(+), and neurospheres U87 glioma cell cultures.

**Results:**

We found that CD133, COX-2 and MT1-MMP expression were enhanced when glioma cells were cultured in neurosphere conditions. A CD133(+)-enriched U87 glioma cell population, isolated from parental U87 cells with magnetic cell sorting technology, also grew as neurospheres and showed enhanced COX-2 expression. MT1-MMP gene silencing antagonized COX-2 expression in neurospheres, while overexpression of recombinant MT1-MMP directly triggered COX-2 expression in U87 cells independent from MT1-MMP's catalytic function. COX-2 induction by MT1-MMP was also validated in wild-type and in NF-κB p65^-/- ^mutant mouse embryonic fibroblasts, but was abrogated in NF-κB1 (p50^-/-^) mutant cells.

**Conclusion:**

We provide evidence for enhanced COX-2 expression in CD133(+) glioma cells, and direct cell-based evidence of NF-κB-mediated COX-2 regulation by MT1-MMP. The biological significance of such checkpoint control may account for COX-2-dependent mechanisms of inflammatory balance responsible of therapy resistance phenotype of cancer stem cells.

## Background

Despite significant improvements, current therapies have yet to cure infiltrative gliomas. Therapy resistance is possibly attributable to cancer stem cells (CSC), a small subpopulation of cells within the brain tumor mass responsible for the initiation and maintenance of the tumor [1]. Recently, small populations of CSC in adult and pediatric brain tumors were identified and, once isolated from tumor tissues, formed neurospheres when cultured *in vitro *[2,3]. Based upon their high expression of the neural precursor cell surface marker CD133 (prominin-1), these CSC have been further hypothesized to bear properties such as resistance to apoptosis and resistance to both drugs and ionizing radiation [4,5]. While the brain tissue microenvironmental niche is a prerequisite for expression of the stem cell marker CD133 antigen in brain tumors [6], its expression level is also thought to predict clinical outcome in glioma patients [7,8].

High cyclooxygenase (COX)-2 expression is another condition associated with clinically more aggressive gliomas and is, along with CD133, a strong predictor of poor survival [9,10]. COX-2 is an inducible enzyme responsible for prostaglandin production at sites of inflammation [11,12]. In human glioblastoma, COX-2 performs important functions in tumorigenesis [13] and inhibitors of eicosanoid biosynthesis have been shown to suppress cell proliferation and to promote astrocytic differentiation [14]. Since COX-2 protein is overexpressed in the majority of gliomas, it is therefore considered to be an attractive therapeutic target [15,16]. In fact, enhancement of glioblastoma radioresponse by the selective COX-2 inhibitor celecoxib was recently reported [17]. Paradoxically, the effectiveness of COX-2 inhibitors on glioma cell proliferation and radioresponse enhancement was also found to be independent of COX-2 protein expression [18]. This evidence suggests that alternate signaling molecules are associated to therapy resistance and involved in regulating COX-2 expression. These alternate molecules may possibly become attractive therapeutic targets.

Membrane-type matrix metalloproteinases (MT-MMP) constitute a growing subclass of MMP [19]. While most of the MMP are secreted, the MT-MMP are membrane-associated and a number of these have cytoplasmic domains which are important in cellular signaling [20-22]. MT1-MMP is the best-characterized MT-MMP. In addition to activation of proMMP-2, MT1-MMP displays intrinsic proteolytic activity towards extracellular matrix (ECM) molecules. The increased expression levels of several members of the MMP family have been shown to correlate with the graded level of gliomas, including MT1-MMP. Aside from its classical roles, many new functions of MT1-MMP were recently demonstrated, including a role in PGE_2_-induced angiogenesis [23], platelet-mediated calcium mobilization [24], regulation of cell death/survival bioswitch [22,25], and radioresistance in both glioma cells [26,27] and endothelial cells [28]. Finally, the recent demonstration that MT1-MMP also plays a role in medulloblastoma CD133(+) neurosphere-like formation and increased invasiveness [6] reinforces the need to design new therapeutic strategies that either directly target MT1-MMP functions or its associated signaling functions.

In the present study, we examined whether CD133(+) U87 glioma cells are characterized by increased COX-2 and MT1-MMP expression, and whether a potential MT1-MMP/COX-2 signalling axis might be important with respect to the therapy-resistant phenotype of CSC.

## Methods

### Materials

Sodium dodecylsulfate (SDS) and bovine serum albumin (BSA) were purchased from Sigma (Oakville, ON). Cell culture media was obtained from Invitrogen (Burlington, ON). Electrophoresis reagents were purchased from Bio-Rad (Mississauga, ON). The enhanced chemiluminescence (ECL) reagents were from Amersham Pharmacia Biotech (Baie d'Urfé, QC). Micro bicinchoninic acid protein assay reagents were from Pierce (Rockford, IL). The polyclonal antibodies against CD133 and COX-2, and the monoclonal antibody against GAPDH were purchased from Abcam (Cambridge, MA) and Advanced Immunochemical Inc. (Long Beach, CA) respectively. The polyclonal antibody against MT1-MMP (AB815) was from Chemicon (Temecula, CA). Horseradish peroxidase-conjugated donkey anti-rabbit and anti-mouse IgG secondary antibodies were from Jackson ImmunoResearch Laboratories (West Grove, PA). All other reagents were from Sigma-Aldrich Canada.

### Cell culture and neurosphere-like formation

The human U87 glioblastoma cell line was purchased from American Type Culture Collection (Manassas, VA) and was maintained in Eagle's Minimum Essential Medium (EMEM) containing 10% (v/v) calf serum (CS) (HyClone Laboratories, Logan, UT), 2 mM glutamine, 100 units/ml penicillin and 100 mg/ml streptomycin. Cells were incubated at 37°C with 95% air and 5% CO_2_. Neurosphere-like formation was triggered in a defined serum-free neural stem cell medium [29] containing Ex Vivo 15 (Lonza, Walkersville, MD), 20 ng/ml basic fibroblast growth factor, 20 ng/ml of epidermal growth factor (Wisent, St-Bruno, QC), 20 ng/ml leukemia inhibitory factor (Sigma, Oakville, ON) and 1× neural survival factor-1 (Lonza, Walkersville, MD). Murine L929 (L) cells were maintained as previously described [30]. NF-κB p50^-/- ^and p65^-/- ^immortalized fibroblasts were obtained from Dr David Baltimore (California Institute of Technology, Pasadena, CA, USA).

### Magnetic cell sorting and flow cytometry

Confluent U87 glioma parental cells were harvested with cell dissociation buffer (Hank's based; Invitrogen), centrifuged at 800 × g for 5 min and resuspended in 1× PBS with 0.5% BSA and 2 mM EDTA. Magnetic labeling with 100 μl AC133 (CD133/1) Microbeads per 10^8 ^cells was performed for 30 minutes at 4°C using a CD133 Direct Cell Isolation kit (Miltenyi Biotec, Auburn, CA). Fifty μl of 293C3 (CD133-2)-phycoerythrin (fluorochrome-conjugated mouse monoclonal IgG2b; Miltenyi Biotec) was added for an additional 10 min at 4°C to evaluate the efficiency of magnetic separation by flow cytometry. Magnetic separation was carried out using LS columns and a MACS separator (Miltenyi Biotec) under a biological hood. CD133(+) fractions were eluted by removing the colunm from the magnetic field and using a sterile plunger. Aliquots of CD133(+)-sorted cells were evaluated for purity by flow cytometry with a FACSCalibur machine (BD Biosciences). CD133(+)-sorted cell populations were resuspended in SFM with growth hormones.

### Total RNA isolation and quantitative reverse transcription-polymerase chain reaction (qRT-PCR) analysis

Total RNA was extracted from monolayers or neurosphere-like cells using TRIzol reagent (Life Technologies). For cDNA synthesis, 1 μg total RNA was reverse-transcribed into cDNA using a high capacity cDNA reverse transcription kit (Applied Biosystems, Foster City, CA). cDNA was stored at -80°C until PCR. Gene expression was quantified by real-time quantitative PCR using iQ SYBR Green Supermix (BIO-RAD, Hercules, CA). DNA amplification was carried out using an Icycler iQ5 machine (BIO-RAD, Hercules, CA) and product detection was performed by measuring the binding of the fluorescent dye SYBR Green I to double-stranded DNA. All the primer sets were provided by QIAGEN (Valencia, CA). The relative quantities of target gene mRNA against an internal control, 18S ribosomal RNA, was measured by following a ΔC_T _method. An amplification plot comparing fluorescence signal *vs*. cycle number was drawn. The difference (ΔC_T_) between the mean values in the triplicate samples of target gene and those of 18S ribosomal RNA were calculated by iQ5 Optical System Software version 2.0 (BIO-RAD, Hercules, CA) and the relative quantified value (RQV) was expressed as 2^ΔC^T.

### RNA interference

RNA interference experiments were performed using HiPerFect (QIAGEN, Valencia, CA). A small interfering RNA (siRNA; 20 nM) against MT1-MMP (siMT1-MMP) and mismatch siRNA were synthesized by EZBiolab Inc. (Westfield, IN), and annealed to form duplexes. The sequence of the siMT1-MMP used in this study was derived from the human MT1-MMP gene (NM_004995) and is as follows: 5'-CCAGAAGCUGAAGGUAGAAdTdT-3' (sense) and 5'-UUCUACCUUCAGCUUCUGGdTdT-3' (antisense) [31]. Evaluation of the transient knockdown duration was performed by real-time quantitative RT-PCR and the targeted gene expression was found to be routinely diminished by 65–90% 24 to 48 hrs post-transfection (not shown).

### Cell transfection method

Sub-confluent U87 monolayer cells were transiently transfected with 10 μg of the cDNA encoding full length (Wt) MT1-MMP fused to GFP [22] using Lipofectamine 2000 (Invitrogen, Burlington, ON). Mock transfections of U87 cultures with the empty vector, pcDNA (3.1+), were used as controls. Transfected cells were left to recuperate and were used 48 hrs post-transfection. MT1-MMP specific gene expression and function was evaluated by semi-quantitative RT-PCR and immunoblotting procedures, and was validated by assessing MT1-MMP-mediated proMMP-2 activation by gelatin zymography.

### Gelatin zymography

Gelatin zymography was used to assess the extracellular levels of proMMP-2 and MMP-2 activities. Briefly, an aliquot (20 μl) of the culture medium was subjected to SDS-PAGE in a gel containing 0.1 mg/ml gelatin. The gels were then incubated in 2.5% Triton X-100 and rinsed in nanopure distilled H_2_O. Gels were further incubated at 37°C for 20 hrs in 20 mM NaCl, 5 mM CaCl_2_, 0.02% Brij-35, 50 mM Tris-HCl buffer, pH 7.6 and then stained with 0.1% Coomassie Brilliant blue R-250 and destained in 10% acetic acid, 30% methanol in H_2_O. Gelatinolytic activity was detected as unstained bands on a blue background.

### Immunoblotting procedures

Proteins from control and treated cells were separated by SDS-polyacrylamide gel electrophoresis (PAGE). After electrophoresis, proteins were electrotransferred to polyvinylidene difluoride membranes which were then blocked for 1 hr at room temperature with 5% non-fat dry milk in Tris-buffered saline (150 mM NaCl, 20 mM Tris-HCl, pH 7.5) containing 0.3% Tween-20 (TBST). Membranes were further washed in TBST and incubated with the primary antibodies (1/1,000 dilution) in TBST containing 3% bovine serum albumin, followed by a 1 hr incubation with horseradish peroxidase-conjugated anti-rabbit or anti-mouse IgG (1/2,500 dilution) in TBST containing 5% non-fat dry milk. Immunoreactive material was visualized by enhanced chemiluminescence (Amersham Biosciences, Baie d'Urfée, QC).

## Results

### CD133, COX-2 and MT1-MMP expression is increased in neurosphere-like U87 glioma cultures

**Figure 1:**
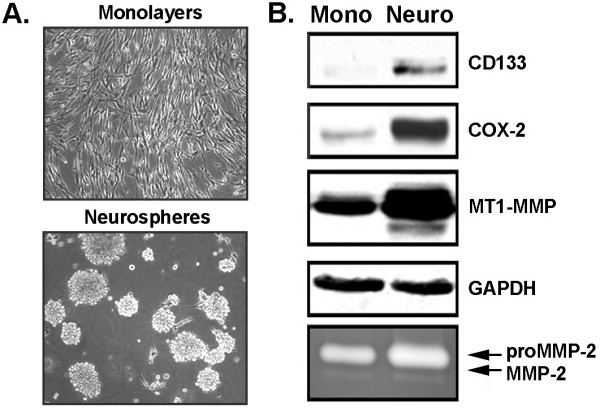
**CD133, COX-2 and MT1-MMP expression are increased in U87 neurospheres glioma cultures**. (A) U87 glioblastoma-derived cell lines were cultured as monolayers or non-adherent neurospheres as described in the Methods section and representative phase contrast photographs were taken. (B) Cell lysates were isolated from U87 glioblastoma-derived cells and SDS-PAGE performed (20 μg protein/well), followed by Western blotting and CD133, COX-2, MT1-MMP or GAPDH immunodetection. Gelatin zymography was also used to monitor the extent of latent proMMP-2 and active MMP-2 expression from the conditioned media of the serum-starved cells.

Neurosphere-like brain CSC are thought to contribute to a sub-population of CD133(+) brain CSC [32]. Neurosphere induction in U87 cells was performed according to established protocols [33,34]. This process promoted the transition of adherent monolayer cells to non-adherent, neurosphere-like cells (Figure 1a). Immunodetection of CD133, COX-2 MT1-MMP and GAPDH was performed on the cell lysates. Neurosphere culture conditions induced CD133 and COX-2 expression in U87 cells when compared to their corresponding monolayer cultures (Figure 1b). MT1-MMP expression was also induced during neurosphere-like formation, while the house-keeping gene GAPDH remained unaffected (Figure 1b). Since MT1-MMP is known to activate proMMP-2 into MMP-2, the levels of latent proMMP-2 and active MMP-2 were also assessed in those same serum-starved monolayer and neurosphere culture conditions. We observed that proMMP-2 activation in U87 neurospheres remained unchanged. Therefore, increased MT1-MMP seems to occur independent of its capacity to induce proMMP-2 activation, suggesting that MT1-MMP may regulate alternate intracellular processes. Collectively, increased CD133, COX-2 and MT1-MMP expression characterizes neurosphere-like formation in U87 glioma cells.

### Cell-based evidence that MT1-MMP directly regulates COX-2 expression in U87 glioma cell lines

**Figure 2:**
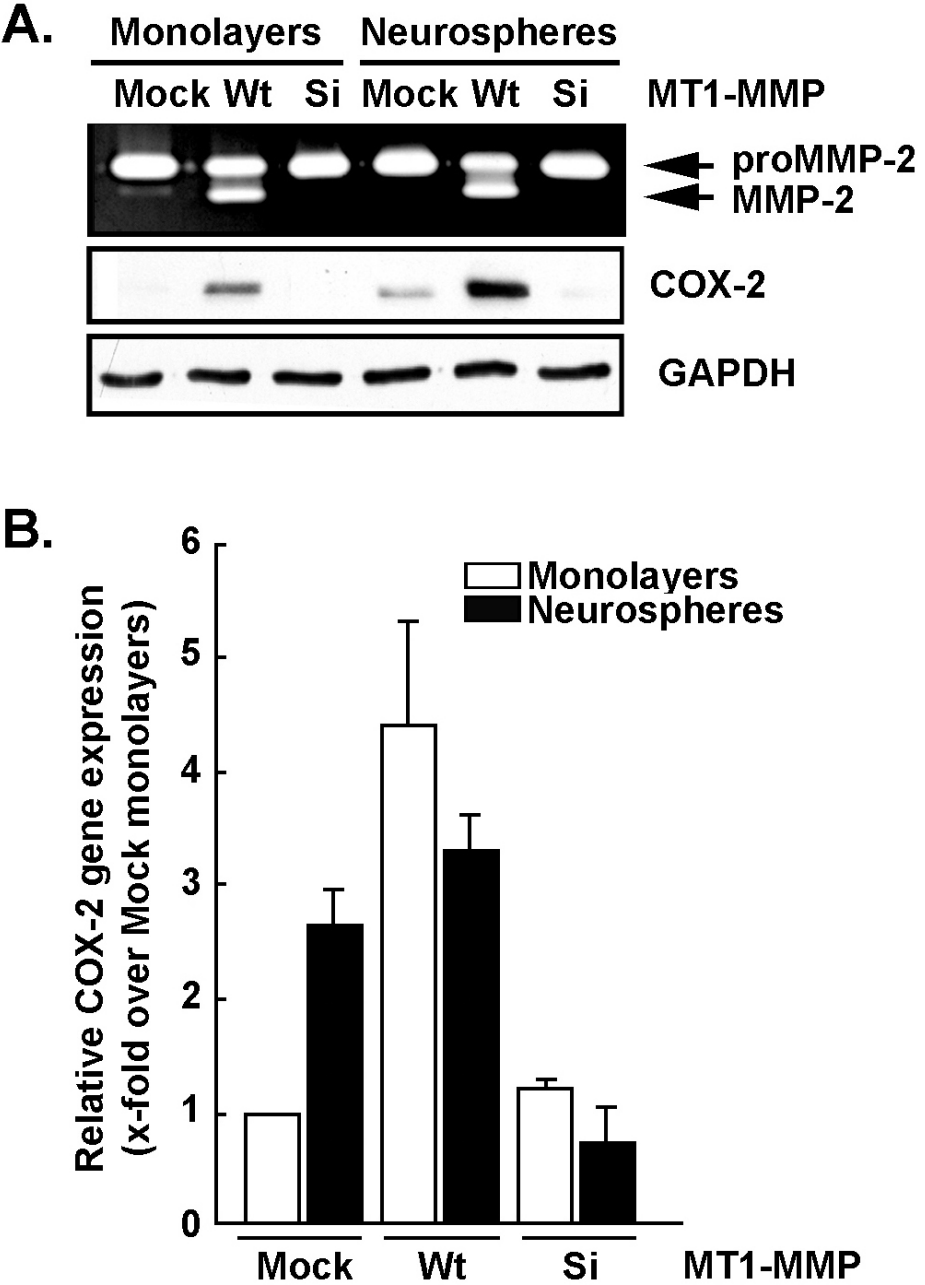
**Cell-based evidence that MT1-MMP directly regulates COX-2 expression in U87 glioma cell lines**. Monolayers or neurospheres from glioblastoma-derived cells were either Mock-transfected, transfected with a cDNA plasmid encoding MT1-MMP (Wt), or transfected with an siRNA (Si) against MT1-MMP as described in the Methods section. (A) Gelatin zymography was performed to monitor the extent of latent proMMP-2 and active MMP-2 expression from the conditioned media of the serum-starved cells. Cell lysates were isolated from U87 glioblastoma-derived cells and SDS-PAGE performed (20 μg protein/well), followed by Western-blotting and COX-2 or GAPDH immunodetection. (B) Total RNA was isolated from monolayers (white bars) or neurospheres (black bars) of U87 Mock-transfected cells, or from U87 cells transfected with MT1-MMP cDNA or siRNA against MT1-MMP, and reverse-transcribed as described in the Methods section. Quantitative PCR was performed in order to monitor COX-2 gene expression levels.

In light of the correlation between MT1-MMP and COX-2 expression observed in U87 glioma neurospheres, we next sought to assess whether MT1-MMP regulates COX-2 expression. U87 cell monolayers were transiently transfected with either a cDNA plasmid encoding recombinant MT1-MMP or with siRNA against MT1-MMP. Cells were then trypsinized and cultured as monolayers or neurospheres as described in the Methods section. Conditioned media from serum-starved cells was harvested in order to monitor the extent of secreted proMMP-2 and MMP-2 levels by gelatin zymography and cell lysates were used for COX-2 and GAPDH immunoblotting. We found that MT1-MMP was effectively overexpressed under all conditions as it triggered proMMP-2 activation into MMP-2 (Figure 2a, upper panel). When COX-2 protein levels were assessed under those same experimental conditions, we found that overexpression of MT1-MMP also triggered COX-2 expression (Figure 2a, middle panel). While basal neurosphere culture conditions re-confirmed COX-2 expression in U87 cells, the neurosphere culture conditions in which MT1-MMP gene expression was downregulated (siMT1-MMP) were not associated with increased COX-2 expression (Figure 2a, middle panel). COX-2 gene expression levels were also assessed by qRT-PCR as described in the methods section using total RNA isolated from U87 monolayers and neurospheres cultures treated as in Figure 2a. We observed a good correlation between COX-2 gene and protein expression (Figure 2b) suggesting that COX-2 transcriptional regulation is involved during neurospheres formation and that this is performed through an MT1-MMP-mediated signaling.

### CD133-sorted U87 glioma cells grow as neurospheres and express high levels of COX-2

**Figure 3:**
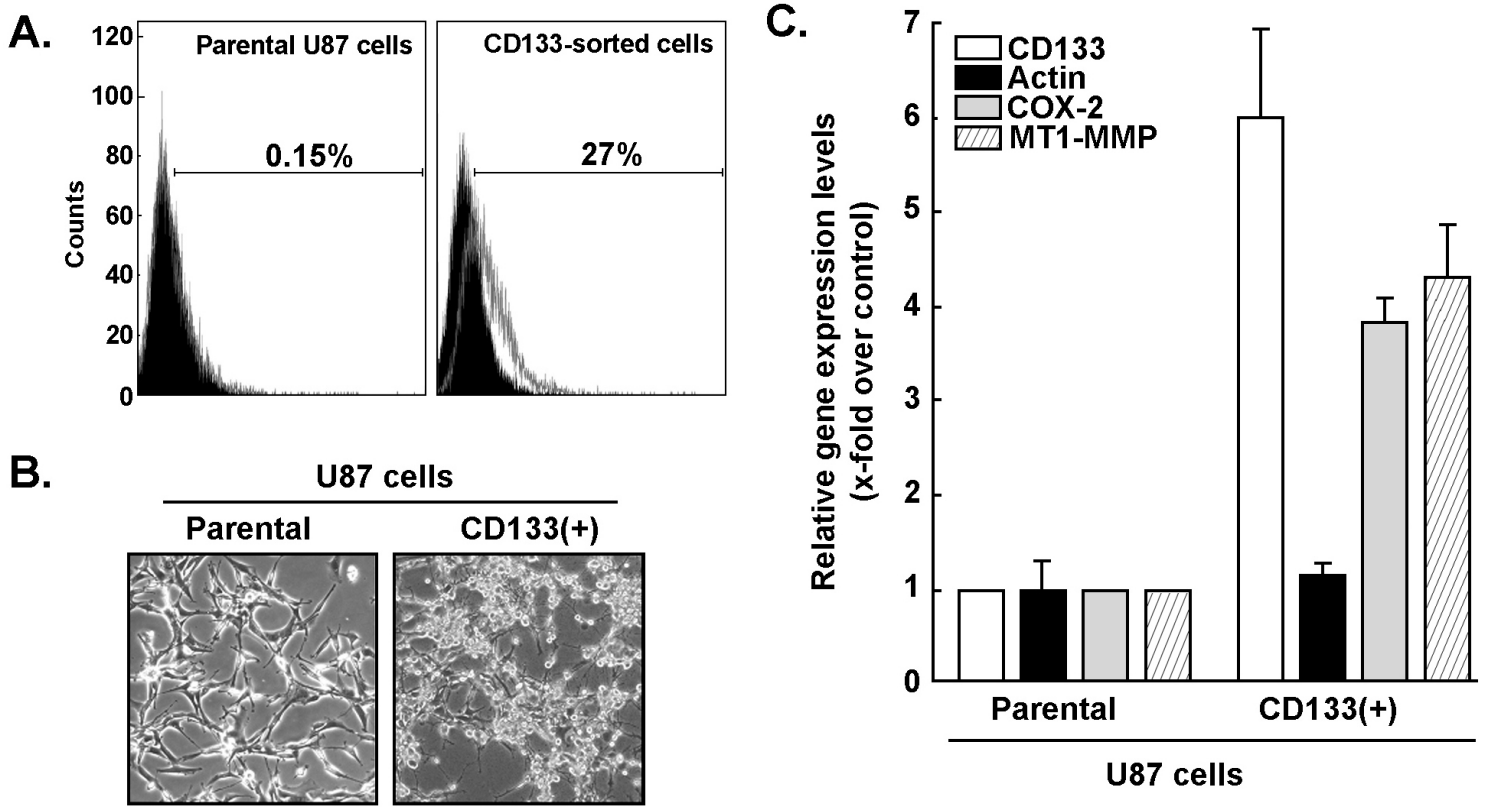
**CD133-sorted U87 glioma cells grow as neurospheres and express high levels of COX-2**. (A) U87 CD133(+) cells were isolated from the parental U87 cells as described in the Methods section using MACS technology. Evaluation of CD133 cell surface expression was then performed by flow cytometry on parental U87 and CD133(+) sorted cells. (B) U87 CD133(+) cells were put back into culture and show a typical neurosphere phenotype, unlike their parental counterpart. (C) Total RNA was extracted from parental U87 and CD133(+) U87 cells and gene expression levels were assessed by qRT-PCR for CD133 (white bars), β-actin (black bars), MT1-MMP (lined bars) and COX-2 (grey bars).

In order to evaluate the potential contribution of the CD133(+) cell subpopulation to the MT1-MMP/COX-2 signalling axis, we used magnetic cell sorting (MACS) technology to isolate CD133(+) cells from the parental U87 glioma cell population. We found that the CD133(+) U87 cell population represented ~0.15% of the total parental U87 glioma cells (Figure 3a, left panel). Sorting of the CD133(+) cells was then performed and we evaluated the cells as being ~27% CD133 positive (Figure 3a, right panel). The isolated subpopulation, with an enrichment of ~180-fold for CD133(+) U87 cells, was put into culture. Cell morphologies of the parental and CD133(+) U87 glioma cells were compared and we observed that the CD133(+) cells formed spontaneous neurospheres (Figure 3b), a characteristic of brain CSC in agreement with previous reports [33,35]. Total RNA was isolated from both parental and CD133(+) glioma cells in order to assess gene expression levels of CD133, COX-2, and β-Actin. We found that CD133 gene expression was increased by ~6-fold in the sorted CD133(+) U87 glioma cells (Figure 3c), in agreement with the increased CD133 cell surface expression (Figure 3a). Moreover, MT1-MMP and COX-2 gene expression were also increased by ~4-fold in CD133(+) U87 cells (Figure 3c).

### MT1-MMP-mediated regulation of COX-2 expression is independent of MT1-MMP's catalytic functions

**Figure 4:**
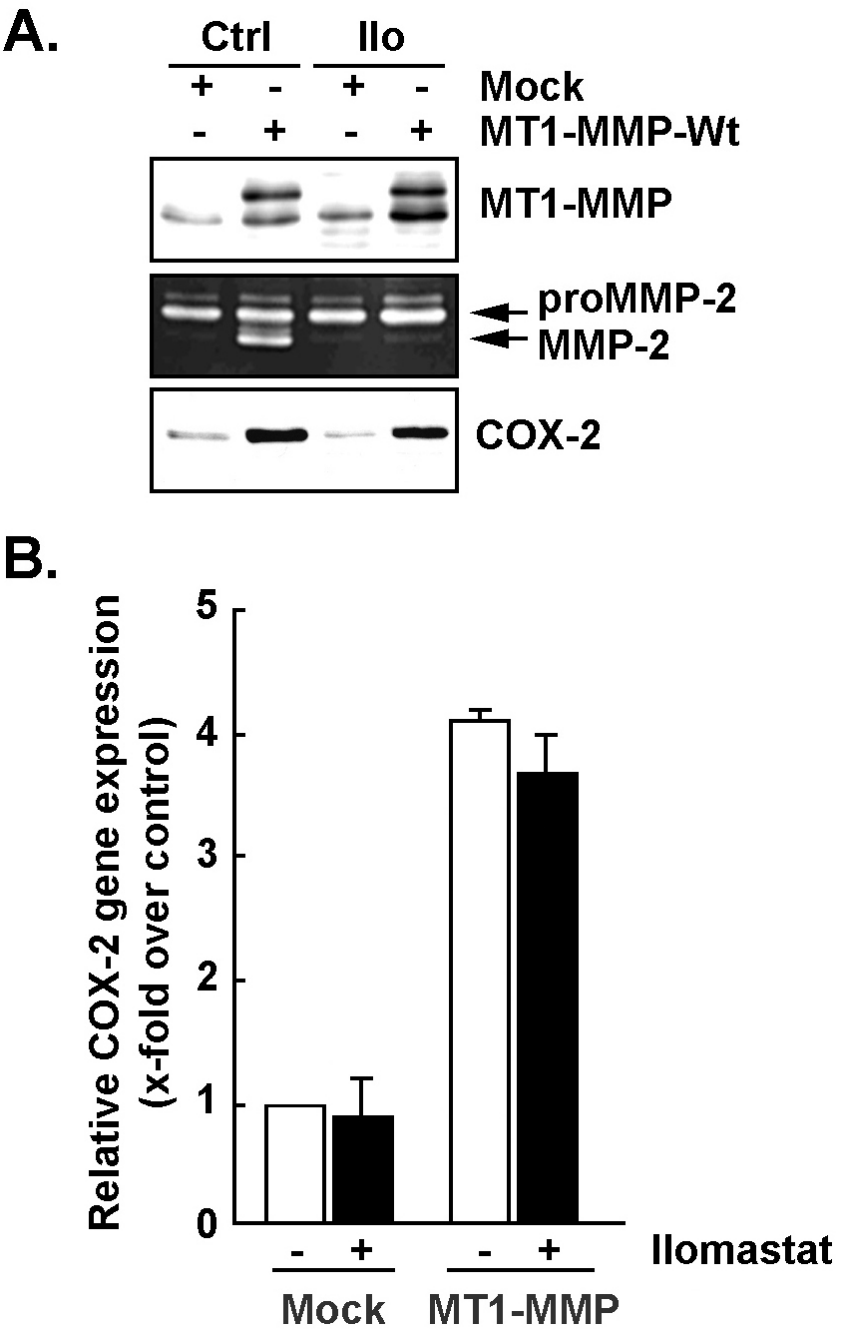
**MT1-MMP-mediated regulation of COX-2 gene and protein expression is independent from MT1-MMP's catalytic functions**. (A) Cell lysates were isolated from Mock-transfected, or U87 glioma cells that had been transiently transfected with a cDNA plasmid encoding MT1-MMP, which were subsequently treated (or not) with 10 μM Ilomastat (Ilo). SDS-PAGE was performed (20 μg protein/well), followed by Western-blotting and COX-2 or MT1-MMP immunodetection. Gelatin zymography was also used to monitor the extent of latent proMMP-2 and active MMP-2 expression from the conditioned media of the serum-starved cells. (B) Total RNA was extracted from the above described cell conditions and gene expression levels assessed by qRT-PCR for COX-2 in the absence (white bars) or the presence of Ilomastat (black bars).

In order to investigate the molecular mechanism involved in MT1-MMP's regulation of COX-2, we first assessed the implication of its catalytic function. U87 glioma cells were transfected with a cDNA encoding MT1-MMP, and then treated (or not) with Ilomastat, a broad-spectrum MMP catalytic inhibitor. Transfection efficacy was confirmed by the appearance of the recombinant MT1-MMP protein by Western blotting (Figure 4a, upper panel). Gelatin zymography further confirmed the appropriate targeting of MT1-MMP to the cell surface since its extracellular catalytic domain triggered proMMP-2 conversion into its active MMP-2 form (Figure 4a, middle panel). As expected, treatment of MT1-MMP-transfected cells with Ilomastat abrogated proMMP-2 activation (Figure 4a, middle panel). COX-2 expression was induced by MT1-MMP overexpression, but was insensitive to Ilomastat's inhibition of cell surface MT1-MMP activity (Figure 4a, lower panel). These results suggest that MT1-MMP's extracellular catalytic functions are not required for inducing COX-2 expression and necessitate an alternative intracellular signaling mechanism triggered by MT1-MMP's intracellular domain. Accordingly, overexpression of a cytoplasmic domain-deleted MT1-MMP [20,21] was unable to trigger COX-2 expression (not shown). Total RNA was next isolated and COX-2 transcriptional regulation assessed upon MT1-MMP overexpression in U87 cells. We observed that transfected cells overexpressing MT1-MMP had significantly elevated levels of COX-2 transcripts (Figure 4b). The possible involvement of nuclear factor kappaB (NF-κB)-intracellular signaling in MT1-MMP-mediated COX-2 transcriptional regulation was next considered.

### COX-2 induction by MT1-MMP occurs through NF-κB-mediated mechanisms

**Figure 5:**
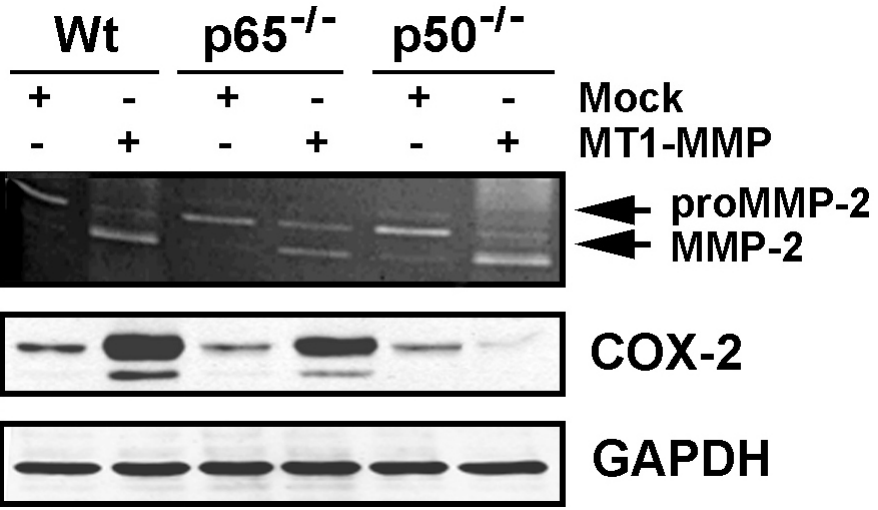
**COX-2 induction by MT1-MMP occurs through NF-κB-mediated mechanisms**. Wild-type (Wt), p65^-/-^, and p50^-/- ^mouse embryonic fibroblasts (MEF) were Mock-transfected or transfected with a cDNA plasmid encoding MT1-MMP. Gelatin zymography was used to monitor the extent of latent proMMP-2 and active MMP-2 expression from the conditioned media of the serum-starved cells (upper panel). Cell lysates were isolated and SDS-PAGE performed (20 μg protein/well), followed by Western blotting and COX-2 immunodetection.

MT1-MMP was previously demonstrated to possess the ability to trigger intracellular signaling through its 20 amino acid intracellular domain [20-22]. Moreover, COX-2 transcriptional expression is thought to be regulated, in part, through NF-κB-mediated signaling involving nuclear translocation of the NF-κB heterodimer p50:p65 [36]. Wild-type mouse embryonic fibroblasts (MEF) as well as p50^-/- ^and p65^-/- ^NF-κB mutants were used to assess MT1-MMP involvement in COX-2 expression. Cell lysates as well as conditioned media were isolated from Mock-transfected and MT1-MMP-transfected cells. Expression and cell surface activity of the recombinant MT1-MMP were confirmed in transfected cells as Wt, p50^-/- ^and p65^-/- ^cells all exhibited increased proMMP-2 activation into its active MMP-2 form as judged by gelatin zymography (Figure 5, upper panel). When COX-2 protein expression was assessed, we observed the induction of COX-2 by MT1-MMP in Wt-MEF (Figure 5, middle panel) confirming the results observed in U87 glioma cells (Figure 4a). Similar MT1-MMP-mediated COX-2 induction was also observed in p65^-/- ^mutant MEF but COX-2 expression was completely abrogated in p50^-/- ^mutant MEF (Figure 5, middle panel). This cell-based evidence directly demonstrates the specific involvement of p50 in NF-κB-mediated MT1-MMP regulation of COX-2 expression.

## Discussion

Overexpression of COX-2, the enzyme responsible for the synthesis of prostaglandin subtype PGE_2_, has been found to be important in the development of several human tumor types such as colon, gastric, pancreatic, and brain tumors [9,37], and has also been associated with high tumor aggressiveness and poor patient prognosis [15,38]. In cell studies, the growth rate of glioblastoma multiforme (GBM) cells correlated with the level of COX-2 enzyme expression, and PGE_2 _is thought to inhibit these cells' apoptosis [39] and to act as a radioprotector [40,41]. As GBM is a high-grade primary brain tumor that is refractory to current forms of treatment possibly due to the presence of tumor-derived CSC, such molecular and cellular attributes may therefore reflect the CSC therapy resistance phenotype. In light of our results showing increased expression of COX-2 in CD133(+) U87-derived glioma cells as well as in CD133-enriched U87 neurospheres, it could be hypothesized that this molecular signature may, in part, be responsible for the therapy resistance phenotype attributable to CSC.

Membrane-type matrix metalloproteinases (MT-MMP) constitute a growing subclass of MMP, with MT1-MMP being the best-characterized MT-MMP whose expression correlates with high-grade gliomas [19]. Aside from its well-established roles in the activation of proMMP-2 and its intrinsic proteolytic activity towards ECM molecules, many new functions of MT1-MMP have recently been demonstrated which include a role in PGE_2_-induced angiogenesis [23] as well as radioresistance in glioma cells [26,27]. The recent demonstration that MT1-MMP also plays a role in medulloblastoma CD133(+) neurosphere-like formation and increased invasiveness [6] further supports the concept of a molecular interplay between MT1-MMP and COX-2. Besides glioblastoma cells, such a link has also been observed in cells derived from malignant fibrous histiocytoma, one of the highest-grade sarcomas arising in bone and soft tissue, where concomitant increased levels of expression of COX-2 and of MT1-MMP were described [42]. Overexpression of COX-2 was also found to elevate tumorigenicity, tumor growth and invasion of human KB carcinoma cells via up-regulated MT1-MMP activity [43]. Finally, co-distribution of MT1-MMP, MMP-2 and COX-2 was demonstrated in grade IV atheroma, again indicating a possible link between these enzymes in the destabilization of atherosclerotic plaques [44]. Altogether, these published data suggest a molecular signaling convergence linking COX-2 to MT1-MMP expression.

By virtue of its ability to regulate the expression of genes involved in cell apoptosis, differentiation, adhesion, and survival, NF-κB constitutes the point of convergence of many oncogenic pathways [45]. Aside from its critical role in the development of human cancer, NF-κB has also been implicated at the molecular level in the promotion of angiogenesis, which is of particular interest since malignant astrocytomas are highly vascular tumors [46]. NF-κB is also a transcriptional regulator of inducible expression of genes including COX-2 [47]. Interestingly, a consensus binding site for NF-κB p65 (TGGAGCTTCC) was found in the 5'-flanking region of the human MT1-MMP gene [48] and NF-κB-mediated induction of MT1-MMP was confirmed in murine melanoma cells [49] and in human fibrosarcoma cells [50]. Further studies also implicated NF-κB as a potentially critical factor in astrocytic tumorigenesis and astrocytoma progression through analysis of cell lines and preclinical models [51-53]. NF-κB functions as a hetero- or homo-dimer which can be formed from five NF-κB subunits, NF-κB1 (p50 and its precursor p105), NF-κB2 (p52 and its precursor p100), RelA (p65), RelB and c-Rel. The most studied dimer, p50:p65, is activated by the classical pathway and usually promotes gene expression. In the current study, we provide evidence for a MT1-MMP-mediated signaling cascade that leads to activation of COX-2 expression that is independent of MT1-MMP's catalytic function (Figure 4). We also demonstrate that this new MT1-MMP/COX-2 signaling axis, in fact, absolutely requires NF-κB p50 while knockdown of NF-κB p65 still enabled MT1-MMP to trigger COX-2 expression. In support to our results, an increase in NF-κB p50 was recently found to rapidly induce MT1-MMP expression in trabecular meshwork cells [54]. Given MT1-MMP's well documented roles in actin/tubulin cytoskeleton perturbation associated to cell migration or tubulogenesis processes, very exciting and recent studies documented a new and underestimated role for dynein/dynactin complex in the nuclear translocation of NF-κB [55]. Whether such microtubule involvement is affected by MT1-MMP and that would mediate specific p50 nuclear translocation certainly deserves further experimentation.

Among the therapeutic molecules that could be envisioned to target COX-2 functions in CSC, the radiosensitizing actions of meloxicam and celecoxib may be considered in light of their inhibition of PGE_2 _production [18,56]. Future experimental studies on the growth inhibitory and radiosensitizing effects of these two molecules should focus on PGE_2_synthesis and on apoptosis induction in CSC. In fact, our present findings that COX-2 induction correlates with CD133 expression in human glioma cell lines demonstrates that selective COX-2 inhibitors may thus yield a promising perspective to further improve the therapy of glioma patients. Therefore, the development of pharmaceutical approaches that alter expression of MT1-MMP or the MT1-MMP/COX-2 signaling axis in neuroinflammation provides new biological significance that prompts in targeting invading glioma cells.

## Abbreviations

COX: cyclooxygenase; CSC: cancer stem cells; ECM: extracellular matrix; MEF: mouse embryonic fibroblasts; MT1-MMP: membrane type-1 matrix metalloproteinase; NF-κB: nuclear factor kappaB.

## Competing interests

The authors declare that they have no competing interests. None of the authors hold stocks or shares in any pharmaceutical company or hold or are applying for any patents relating to the contents of the manuscript.

## Authors' contributions

BA has conceived, designed, analyzed and interpreted the data of this study. CL has acquired, analyzed, and was involved in drafting the manuscript. AS and MPL have acquired the data. RB has conceived, designed and supported financially this study. All authors read and approved the final version of this manuscript.
